# A Comparison of Airway Ultrasound and Clinical Parameters for Predicting Difficult Laryngoscopy in the Emergency Department: A Study Protocol

**DOI:** 10.7759/cureus.69543

**Published:** 2024-09-16

**Authors:** Shubhada Kadadi, Charuta Gadkari, Aditya Pundkar

**Affiliations:** 1 Department of Emergency Medicine, Jawaharlal Nehru Medical College, Datta Meghe Institute of Higher Education and Research, Wardha, IND; 2 Department of Orthopedics, Jawaharlal Nehru Medical College, Datta Meghe Institute of Higher Education and Research, Wardha, IND

**Keywords:** cormack-lehane, emergency, laryngoscope, lemon criteria, ultrasound

## Abstract

Background: Airway assessment plays an important part in airway management. Over the years, ultrasound has emerged as an alternative modality for airway assessment. We aim to assess the accuracy of clinical parameters, ultrasonographic upper airway parameters, and laryngoscopic grade, using the Cormack-Lehane classification, to predict difficult laryngoscopy in the emergency department (ED). The primary objective is to determine the accuracy of ultrasonography parameters compared to Cormack-Lehane grading in predicting difficult laryngoscopy in adults. The secondary objective is to assess the accuracy of clinical parameters compared to Cormack-Lehane grading in predicting difficult laryngoscopy in adults.

Methods: A sample of 62 adult patients over 18 years who come to the emergency department and require endotracheal intubation will undergo an ultrasonographic examination of the upper airway. During this examination, an emergency medicine physician will measure the skin-to-epiglottis distance and tongue thickness using the GE Healthcare (Chicago, IL) ultrasound and color Doppler (model: Logic E).

Results: The results of the study will be drawn in 2025 upon its completion.

Conclusions: The conclusion will be drawn regarding the correlation between ultrasound parameters and Cormack-Lehane grading and clinical parameters in predicting difficult laryngoscopy.

## Introduction

Effective airway management is crucial for patients in critical conditions. However, it remains a challenge in the emergency department (ED). Loss of control of the airway can lead to catastrophic effects. Airway assessment plays an important part in airway management [1]. Predicting difficult airways is often hampered in the ED. Traditionally used clinical parameters such as Look externally, Evaluate the 3-3-2 rule, Mallampati score, Obstructed airway, and Neck mobility (LEMON) have limitations in emergency settings due to factors like uncooperative patients, cervical spine immobilization, and obtunded patients [2]. Ultrasound has emerged as an alternative modality for airway assessment in recent years, as it is a mobile, noninvasive, and nonionizing technology [3].

Ultrasound allows real-time visualization of anatomical structures, aiding in rapid assessment. It helps gauge parameters like airway patency, identify potential obstructions, and assess the position of vocal cords. This dynamic imaging enhances decision-making during interventions such as intubation through optimal preparation involving appropriate choice of equipment and personnel, making the process more precise and reducing complications [4].

The Cormack-Lehane classification is the gold standard for defining difficult laryngoscopy. Hence, airway assessment parameters have classically been compared to the Cormack-Lehane classification [5]. The literature search revealed multiple studies comparing clinical assessment indices versus ultrasound parameters in preoperative settings. However, studies in emergency settings are limited [6]. This research delves into the comprehensive use of ultrasound in airway management in emergency settings through clinical parameters like LEMON criteria, ultrasound measurements like skin-to-epiglottis distance, tongue thickness, and Cormack-Lehane classification on direct laryngoscopy as key metrics.

## Materials and methods

Study design and setting

This will be a prospective cross-sectional study of adult patients coming to the ED requiring endotracheal intubation. After obtaining ethical and research clearance from the Ethics Committee at the Datta Meghe Institute of Higher Education and Research, the study will be conducted in Acharya Vinoba Bhave Rural Hospital. Informed consent will be obtained from study participants. A trained Emergency Medicine physician unaware of the clinical and ultrasound airway parameters will perform a laryngoscopy and record Cormack-Lehane grading.

Inclusion criteria

Patients 18 years old and older who need endotracheal intubation will be included.

Exclusion criteria

Patients with limited mouth opening, head and neck pathologies, and upper airway anatomical anomalies, trauma, or tumors will be excluded. Patients who have undergone tracheostomy, pregnant patients, and those with limited neck extension will be excluded from the study.

Statistical analysis

A sample size of 62 is derived based on the article by Sotoodehnia et al. [1]. The estimated proportion with absolute precision is as follows: alpha (α) = 0.05, estimated proportion (p) = 0.799, and estimation error (d) = 0.10.

Procedure

Clinical airway assessment of patients in a supine position will be done using LEMON criteria, and findings will be noted. All enrolled patients will undergo a duly explained ultrasonographic examination of the upper airway. Ultrasound-guided parameters will be measured using GE Healthcare (Chicago, IL) ultrasound and color Doppler (model: Logic E) curvilinear transducer. The parameters like skin-to-epiglottis distance and tongue thickness will be measured. To begin with, the probe will be placed in the midline, and then the neck scanning will be done in the transverse plane, scanning plane sections through both the vocal cords, from the cephalad to the caudal direction. The thickness of the anterior soft tissue will be measured at the level of the vocal cord, from the skin to the epiglottis. Tongue thickness will be defined as the maximum vertical length measured from the tongue surface to the submental skin.

Ethical approval

Approval is sought from the Ethical Committee of the Datta Meghe Institute of Higher Education and Research under approval no. DMIHER(DU)/IEC/2024/193 and the Clinical Trials Registry of India (CTRI) under CTRI no. CTRI/2024/06/069097. Written informed consent will be obtained from all patients included in the study.

## Results

Data will be compiled in an Excel sheet (Microsoft Corporation, Redmond, WA) and analyzed using appropriate statistical tests. The results will be drawn in 2025 after the study is completed. A positive impact is expected, as ultrasound will become an essential tool in emergency airway management, enhancing patient safety and clinical outcomes.

## Discussion

Traditionally, various clinical parameters such as thyromental distance, Mallampati score, and neck circumference have been employed to anticipate challenging airways. However, these methods while useful have limitations in emergent situations where rapid and accurate assessment is paramount [7-10]. This study evaluates the comparative effectiveness of airway ultrasound versus conventional clinical parameters in predicting difficult laryngoscopy within an ED setting [3,11,12]. Airway ultrasound has demonstrated significant potential over time due to its noninvasiveness, real-time imaging capabilities, and its ability to visualize anatomical structures that may not be readily accessible through clinical examination alone. Several studies have highlighted the potential of ultrasound parameters such as the distance from the skin to the epiglottis, the anterior neck soft tissue thickness at the level of the hyoid bone and vocal cords, and the visibility of the hyoid bone in predicting difficult laryngoscopy. These parameters can provide detailed insights into airway anatomy, offering an additional layer of assessment that may enhance the accuracy of predicting a difficult airway [13].

This research will evaluate both airway ultrasound and traditional clinical examination parameters in a cohort of patients presenting to the ED in a prospective cross-sectional study design. Participants will undergo a comprehensive assessment that includes both methods before laryngoscopy. The outcomes of these assessments will then be compared to the actual laryngoscopy grade, which serves as the gold standard for determining airway difficulty [14,15].

One of the primary advantages of using a prospective cross-sectional study design is its ability to provide real-time, context-specific data. In the dynamic and often unpredictable environment of the ED, having reliable and immediately applicable predictive tools is essential. By directly comparing ultrasound findings with clinical examination results and correlating them with laryngoscopic outcomes, this study aims to determine the most accurate and practical method for anticipating difficult airways [3].

Preliminary evidence suggests that airway ultrasound may offer superior predictive value compared to traditional clinical parameters. For instance, ultrasound can accurately measure soft tissue thickness and identify anatomical variations associated with difficult laryngoscopy, which might not be evident through clinical examination alone. This capability is particularly beneficial in emergencies where patients may have anatomical challenges or conditions that complicate airway management [16].

Moreover, airway ultrasound can standardize the assessment process, reducing interobserver variability that often affects clinical examination. While clinical parameters rely heavily on the practitioner's experience and skill, ultrasound provides objective measurements that can be consistently reproduced. This standardization can lead to more reliable predictions and better preparation for managing difficult airways [11,17].

If airway ultrasound proves to be more reliable and accurate in predicting difficult laryngoscopy, it could significantly impact clinical practice in the ED. Emergency physicians could incorporate ultrasound into their routine airway assessment protocols, enhancing their ability to quickly identify patients at risk for difficult laryngoscopy. This early identification can lead to improved preparedness, including the use of advanced airway management techniques, alternative intubation strategies, and the involvement of more experienced personnel when necessary [18].

## Conclusions

The conclusion will be drawn regarding correlation between ultrasound parameters and Cormack-Lehane grading as well as clinical parameters in predicting difficult laryngoscopy. The integration of ultrasound into airway assessment protocols could revolutionize the approach to managing airways in the ED, ensuring that patients receive the best possible care even in the most challenging situations. This research underscores the importance of continuous innovation and evaluation in medical practice to improve patient care and outcomes.
